# ARRDC4 regulates enterovirus 71-induced innate immune response by promoting K63 polyubiquitination of MDA5 through TRIM65

**DOI:** 10.1038/cddis.2017.257

**Published:** 2017-06-08

**Authors:** Jun Meng, Zhenyu Yao, Yaqing He, Renli Zhang, Yanwei Zhang, Xiangjie Yao, Hong Yang, Long Chen, Zhen Zhang, Hailong Zhang, Xueqin Bao, Gang Hu, Tangchun Wu, Jinquan Cheng

**Affiliations:** 1 Department of Microbiology, Shenzhen Center for Disease Control and Prevention, Shenzhen, Guangdong, China; 2 Department of Occupational and Environmental Health Key Laboratory of Environment and Health, Ministry of Education and State Key Laboratory of Environmental Health (Incubating), School of Public Health, Tongji Medical College, Huazhong University of Science and Technology, Wuhan, Hubei, China; 3 Department of Translational Medicine R&D Center, Shenzhen Institute of Advanced Technology, Chinese Academy of Sciences, Shenzhen, Guangdong, China; 4 Department of Epidemiology and Biostatistics, College of Public Health, Zhengzhou University, Zhengzhou, Henan, China; 5 Department of Biostatistics and Epidemiology, College of Public Health, Sun Yat-Sen University, Guangzhou, Guangdong, China

## Abstract

Enterovirus 71 (EV71) is the main causative agent of hand, foot and mouth disease (HFMD), which induces significantly elevated levels of cytokines and chemokines, leading to local or system inflammation and severe complications, whereas the underlying regulatory mechanisms and the inflammatory pathogenesis remain elusive. ARRDC4 is one member of arrestins family, having important roles in glucose metabolism and G-protein-coupled receptors (GPCRs) related physiological and pathological processes, however, the function of ARRDC4 in innate immune system is largely unknown. Here we identified that ARRDC4 expression was increased after EV71 infection in THP-1-derived macrophages and verified in EV71-infected HFMD patients and the healthy candidates. The expression level of ARRDC4 was positively correlated with the serum concentration of IL-6, TNF-*α* and CCL3 in clinical specimens. ARRDC4 interacted with MDA5 via the arrestin-like N domain, and further recruited TRIM65 to enhance the K63 ubiquitination of MDA5, resulting in activation of the downstream innate signaling pathway and transcription of proinflammatory cytokines during EV71 infection. Our data highlight new function of ARRDC4 in innate immunity, contributing to the better understanding about regulation of MDA5 activation after EV71 infection, and also suggest ARRDC4 may serve as a potential target for intervention of EV71-induced inflammatory response.

Enterovirus 71 (EV71) is a single positive-stranded RNA virus belonging to the picornaviridae family. EV71 is the main causative agent of hand, foot and mouth disease (HFMD), which induces a wide range of clinical manifestations leading to severe neurological complications in infants and children younger than 5 years old.^1^ EV71-infected patients carry high level of cytokines and chemokines,^2, 3^ whereas exhibit impaired production of type I interferon (IFN).^4, 5^ In the absence of type I IFNs, the other cytokines and chemokines are irreplaceable to defense against EV71 invasion, however, the disordered production of inflammation mediators may lead to cell or tissue injury. The detailed regulation mechanism and the inflammatory pathogenesis after EV71 infection remain elusive.

Once viruses invade the host, the innate immune system detects viral nucleic acid with pattern recognition receptors (PRRs), including Toll-like receptors (TLRs), retinoic acid-inducible gene-I (RIG-I)-like receptors (RLRs) and other cytosolic sensors.^6^ The crucial members of RLRs, RIG-I and melanoma differentiation-associated gene 5 (MDA5), have non-redundant roles in cytosolic viral RNA sensing.^7^ MDA5 prefers to detect long fragments viral RNA (>4kb) mainly from picornavirus and paramyxovirus.^8, 9, 10^ Potentiated by E3 ligase-mediated K63 polyubiquitination, RIG-I and MDA5 are activated and subsequently activate the essential adaptor protein mitochondrial antiviral signaling (MAVS). MAVS further recruits downstream adaptors to activated transcription factors IFN regulatory factors (IRFs), NF-*κ*B and activator protein 1 (AP-1), inducing production of proinflammatory cytokines and type I IFNs.^11^

RLR-triggered innate immune signaling is tightly regulated to achieve effective viral clearance without immunopathology. Polyubiquitination is one of the key regulatory mechanisms for RLRs. Upon recognition of viral RNA, RIG-I undergoes K63 polyubiquitination catalyzed by tripartite motif protein 25 or Riplet to activate innate signaling pathway,^12, 13^ whereas ring-finger protein 125 (RNF125) acts as negative regulators and mediates K48-linked polyubiquitination of RIG-I and MDA5, leading to their degradation through proteasome.^14^ The regulation of RIG-I ubiquitination has been extensively investigated,^12, 13, 14, 15, 16, 17, 18^ however, the regulation mechanism of MDA5 ubiquitination is poorly understood.

Arrestins family consists of visual arrestins (S antigen (SAG) and arrestin 3 (ARR3)), *β*-arrestin 1–2 (ARRB1 and ARRB2), and *α*-arrestins (arrestin domain-containing 1–5 (ARRDC1–5) and thioredoxin-interacting protein (TXNIP)), which have essential and versatile roles in the signaling of G-protein-coupled receptors (GPCRs).^19^ In recent years, some members of the arrestins family have been reported involving in more diversiform physical and pathological processes. Much of the concerns are about the *β*-arrestins, especially in immune and inflammatory responses.^20^ *β*-Arrestin can negatively regulate LPS triggered proinflammatory cytokines production,^21^ and have moderates roles in antiviral innate immune response.^22^ ARRDC4 is one member of *α*-arrestins, which has the arrestin-like structure and highly conserved polyproline (PY) motif in the C-terminal tail.^19^ ARRDC4 participates in glucose metabolism,^23^ and exhibits more varied functions through recruiting some E3 ligases to induce ubiquitination of GPCRs.^24^ Interestingly, it has been suggested that *α*-arrestin and *β*-arrestin may hetero-associate and have coordinated or antagonistic functions depending on context,^25^ however, the function of ARRDC4 in immune response is largely unknown, thus deserves fuller exploration.

Herein, we analyzed gene expression profiles in EV71-infected macrophages with GeneChip Microarrays, notably, all of the arrestins family members were up or down regulated, and ARRDC4 exhibited the highest elevated level. In EV71-infected clinical specimens, the expression level of ARDDC4 was positively correlated with the serum concentration of proinflammatory cytokines. ARRDC4 interacted with MDA5 and promoted K63 ubiquitination of MDA5 via TRIM65, consequently enhanced MDA5-dependent signaling pathways, leading to strengthening of cytokines production in anti-EV71 innate immune response.

## Results

### ARRDC4 expression is upregulated in response to EV71 infection

**Figure 1:**
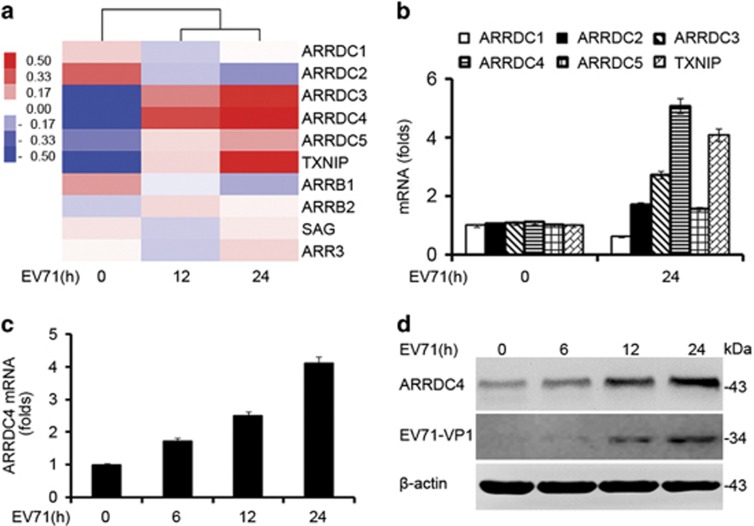
EV71 infection upregulates ARRDC4 expression. (**a**) Microarray analysis of EV71-infected t-M_Ø_ (MOI=50) for indicated time. Heat map represents arrestins family genes expression change (*P*<0.05). (**b**) Q-PCR analysis of expression of *α*-arrestin members in t-M_Ø_ treated with EV71 for 24 h or untreated. (**c**) mRNA expression of ARRDC4 was assayed by Q-PCR in t-M_Ø_ infected with EV71 for indicated time. (**d**) Immunoblot analysis of ARRDC4, EV71-VP1 and *β*-actin expression in lysates of t-M_Ø_ stimulated with EV71 for shown time. Data are shown as mean±S.D. of triplicate samples (**b** and **c**), or are representative of three independent experiments with similar results (**d**)

In order to identify molecules selectively involved in the regulation of EV71-triggered innate immune response, we screened differential expression molecules in EV71-infected THP-1-derived macrophages (t-M_Ø_) through GeneChip Microarrays, and identified all the members of arrestins family were up or downregulated (Figure 1a). ARRDC4 was the most significantly upregulated after EV71 infection (Figure 1a). We validated the expression of *α*-arrestins subfamily members in EV71-infected t-M_Ø_ by Q-PCR, and got the same results as the microarrays (Figure 1b). Moreover, the mRNA and protein levels of ARRDC4 were increased in response to EV71 infection in a time-dependent manner (Figures 1c and d). These results indicate that ARRDC4 may be involved in EV71-induced immune response.

### ARRDC4 promotes EV71-triggered proinflammatory cytokines production

**Figure 2:**
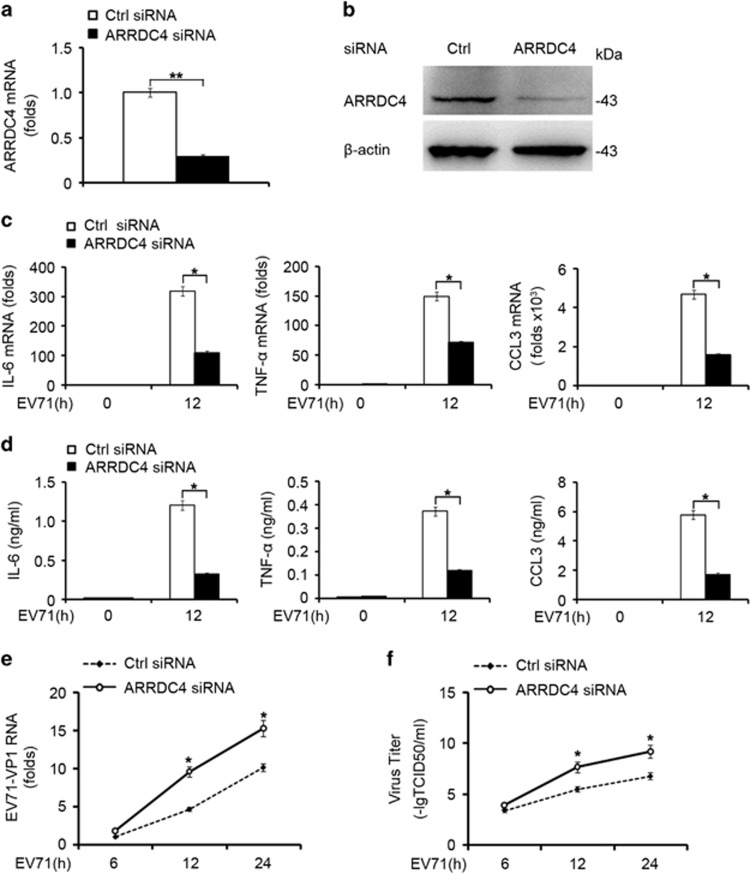
ARRDC4 promotes proinflammatory cytokines production in response to EV71 infection. (**a** and **b**) Q-PCR and immunoblot analysis of ARRDC4 knockdown efficiency in t-M_Ø_ 48 h after transfected with control siRNA or ARRDC4 siRNA. (**c**) Q-PCR analysis of IL-6, TNF-*α* and CCL3 mRNA expression in t-M_Ø_, which were transfected as in (**a**) and infected with EV71 for 12 h. (**d**) ELISA of cytokines in supernatants of t-M_Ø_ treated as in (**c**). (**e**) Q-PCR analysis of EV71-VP1 RNA expression in EV71-infected t-M_Ø_ transfected as in (**a**). (**f**) Tissue culture infective dose (TCID50) assay in supernatants of t-M_Ø_ treated as indicated. Data are shown as mean±S.D. of triplicate samples, *P<0.05 (**a**, **c-f**), or are representative of three independent experiments with similar results (**b**)

To identify the role of ARRDC4 in EV71-triggered immune responses, we silenced ARRDC4 with specific siRNA in t-M_Ø_, and detected significantly knockdown of ARRDC4 expression in both mRNA and protein levels (Figures 2a and b). ARRDC4 silencing antagonized the mRNA expression and the production of IL-6, TNF-*α* and CCL3 in t-M_Ø_ infected with EV71 (Figures 2c and d). However, type I IFN (IFN-*α* and IFN-*β*) expression in mRNA level was inhibited severely by EV71 as reported before,^5^ and knockdown of ARRDC4 did not affect type I IFN production (data not shown). We further observed the antiviral effects of ARRDC4, and found that ARRDC4 silencing promoted EV71 replication in t-M_Ø_ (Figure 2e), accordingly, the viral titer in the supernatant was increased as well (Figure 2f). These data suggest that ARRDC4 positively regulates anti-EV71 innate immune response through promoting IL-6, TNF-*α* and CCL3 production.

### ARRDC4 expression is positively correlated with cytokines production in EV71-infected HFMD patients

**Figure 3:**
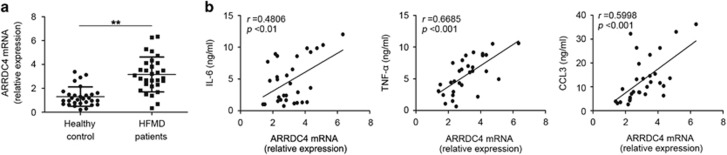
ARRDC4 expression is positively correlated with serum proinflammatory cytokines concentration in EV71-infected HFMD patients and healthy controls. (**a**) Q-PCR analysis of ARRDC4 mRNA expression in PBMCs from EV71-infected HFMD patients (*n*=30) and controls (*n*=30), which normalized by the mean expression value. Data are shown as mean±S.D., **P<0.01. (**b**) The correlation between ARRDC4 mRNA expression level (assayed by Q-PCR) and IL-6, TNF-*α*, CCL3 serum concentration (assayed by ELISA) in patients were evaluated with the Pearson correlation test

We confirmed ARRDC4 expression in peripheral blood mononuclear cells (PBMCs) derived from 30 EV71-infected HFMD children and 30 healthy candidates, which were age (HFMD 38.23±2.880 months, healthy control 40.67±2.156 months, *P*=0.5015) and sex (HFMD 60.0% male, healthy control 56.7% male, *P*=0.7934) matching. The expression levels of ARRDC4 were much higher in patients’ PBMCs compared with control subjects (Figure 3a). We evaluated the correlation between ARRDC4 expression and the serum concentration of proinflammatory cytokines in patients, and found that ARRDC4 mRNA expression level was positively correlated with the concentration of IL-6 (*r*=0.4806, *P*<0.01), TNF-*α* (*r*=0.6685, *P*<0.001) and CCL3 (*r*=0.5998, *P*<0.001) (Figure 3b). These data further verify that ARRDC4 could positively regulate EV71-induced innate proinflammatory cytokines production in HFMD patients.

### ARRDC4 enhances MDA5-triggered signaling activation upon EV71 infection

**Figure 4:**
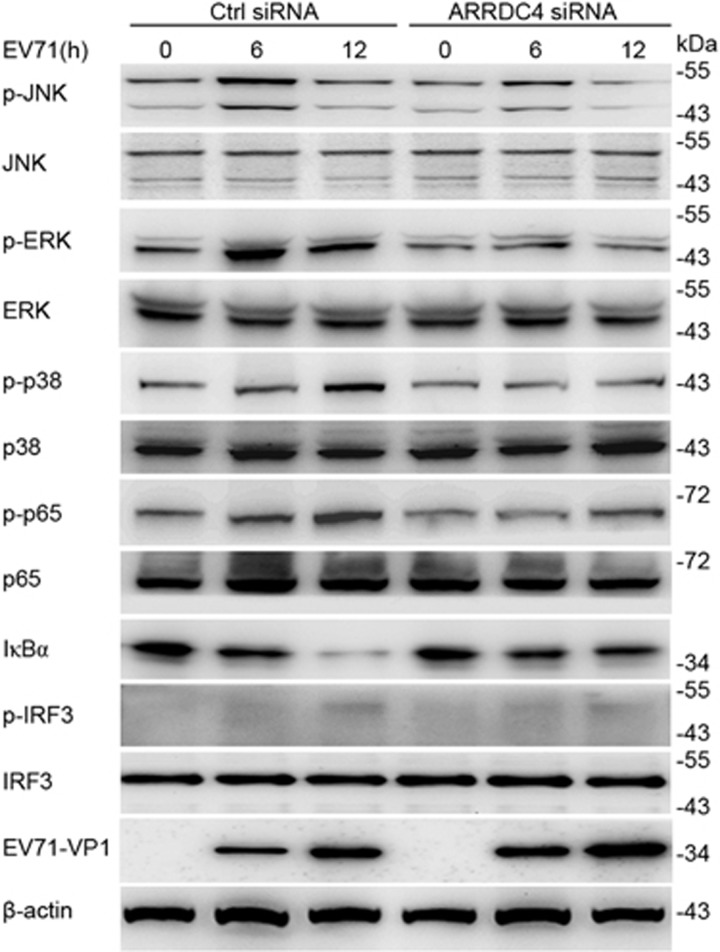
ARRDC4 enhances EV71-triggered activation of NF-*κ*B and MAPK pathway. The t-M_Ø_ were transfected with ARRDC4-specific siRNA or the negative control siRNA. After 48 h, the cells were infected with EV71 for indicated time, then total and phosphor-JNK, -ERK, -p38, -p65, -IRF3 and total I*κ*B*α* were detected with immunoblot, the expression of EV71-VP1 and *β*-actin were detected as well. Data are representative of three independent experiments with similar results

We further investigated the molecular mechanism through which ARRDC4 augmented innate proinflammatory cytokines production. Upon EV71 infection, ARRDC4 silenced t-M_Ø_ exhibited impaired phosphorylation of MAPKs ERK, JNK, p38 and NF-*κ*B subunit p65, and less degradation of I*κ*B*α*, accordingly, the protein level of EV71-VP1 was increased in ARRDC4 silenced t-M_Ø_ (Figure 4). But the activation of IRF3 was weak and similar in both ARRDC4 knockdown and control t-M_Ø_ stimulated with EV71 (Figure 4), which was consistent with the impaired IFN production.^4, 5^ These data demonstrate that ARRDC4 promotes activation of NF-*κ*B and MAPK signaling pathways upon EV71 infection.

The PRRs that recognize EV71 and trigger the activation of innate immune signaling are still elusive. MDA5, which is critical for picornavirus detection, has been reported have important roles during EV71 infection.^26^ In EV71-infected t-M_Ø_, MDA5 silencing significantly inhibited the activation of ERK, JNK, p38, p65, I*κ*B*α* and elevated the protein level of EV71-VP1 (Supplementary Figure S1a and c). On the other hand, the PRRs TLR3 and TLR7/8, which can recognize dsRNA or ssRNA derived from virus, may also participate in EV71-triggered innate immune signaling. Then we knockdown the expression of TLR8 in t-M_Ø_ (Supplementary Figure S1b), and observed TLR8 silencing inhibited the activation of the aforementioned signaling proteins to some extent after EV71 infection, however, the effect was too weakly to promote the expression of EV71-VP1 (Supplementary Figure S1d). Owing to the expression of TLR3 were undetectable in mRNA and protein level in THP-1 cells (data not shown), which was consistent with previous report,^27^ we overexpressed NF-*κ*B luciferase reporter plasmid with TRIF and ARRDC4 in HEK293T cells, and found ARRDC4 did not influence TRIF-induced activation of NF-κB luciferase reporter (Supplementary Figure S1e). These data indicate that, during EV71 infection, MDA5 is the most indispensable PRRs for the activation of innate signaling pathway, whereas the other TLRs may partially have some assistant roles. Collectively, the data above suggest that ARRDC4 promotes EV71-induced inflammatory cytokines production through positively regulation of MDA5-triggered innate signaling activation.

### ARRDC4 interacts with MDA5 via the arrestin-like N domain

**Figure 5:**
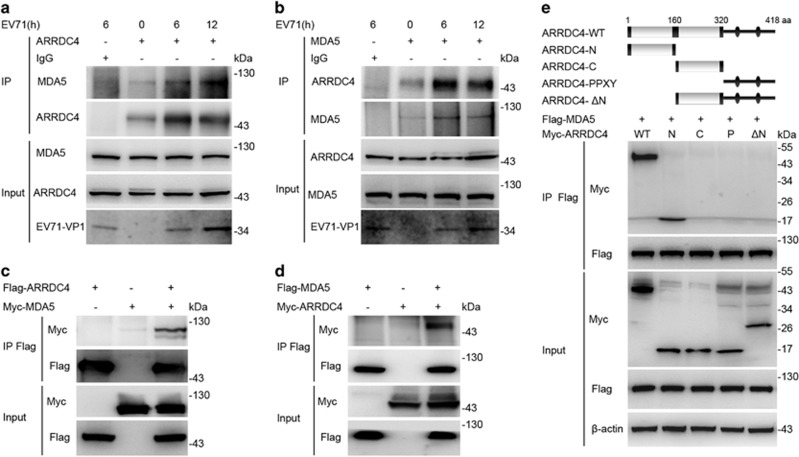
ARRDC4 interacts with MDA5 via arrestin-like N domain. (**a** and **b**) The t-M_Ø_ were infected with EV71, immunoprecipitation (IP) with anti-ARRDC4 (**a**) or anti-MDA5 (**b**) and control IgG antibodies. Immnoblot analysis of the interaction between ARRDC4 and MDA5; input, the whole-cell lysates without immunoprecipitated. (**c** and **d**) HEK293T cells were transfected with Flag- or Myc-tagged ARRDC4 and MDA5 plasmids, after 48 h, IP with anti-Flag antibodies, then detected protein interaction by immunoblot. (**e**) Schematic structure of ARRDC4 and the derivatives were shown, full-length ARRDC4 (ARRDC4-WT), arrestin-like N domain (ARRDC4-N), arrestin-like C domain (ARRDC4-C), PPXY motif (ARRDC4-PPXY). The IP and immunoblot analysis of the interaction between MDA5 and the indicated ARRDC4 constructs in HEK293T cells. Data are representative of three independent experiments with similar results

Given the positive function of ARRDC4 in regulating EV71-induced innate immune signaling, we sought to determine the potential target of ARRDC4. Using immunoprecipitation of endogenous ARRDC4 in EV71-infected t-M_Ø_ plus reverse-phase nanospray liquid chromatography-tandem mass spectrometry assay, we identified ARRDC4 could interacted with MDA5. Then, we corroborated endogenous ARRDC4 could interact with MDA5 in t-M_Ø_ in reciprocal immunoprecipitation experiments (Figures 5a and b). Moreover, we confirmed the interaction between the overexpressed ARRDC4 and MDA5 in HEK293T cells (Figures 5c and d). To determine the domains of ARRDC4 for interaction with MDA5, we constructed various ARRDC4 truncation mutants, and found the full-length ARRDC4 or ARRDC4 with only the arrestin-like N domain (ARRDC4-N) could be co-immunoprecipitated with MDA5 (Figure 5e). The findings indicate that ARRDC4 interacts with MDA5 via the ARRDC4-N domain after EV71 infection.

### ARRDC4 is required for K63 ubiquitination of MDA5 upon EV71 infection

**Figure 6:**
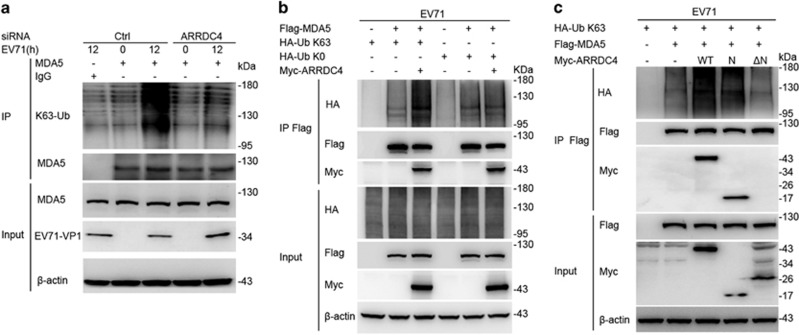
ARRDC4 mediates K63 ubiquitination of MDA5. (**a**) Immunoblot analysis of K63 polyubiquitination of the precipitated MDA5 in t-M_Ø_, which were transfected and stimulated as indicated. (**b** and **c**) HEK293T cells were transfected with indicated plasmids and infected with EV71 for 12 h, followed by IP with anti-Flag antibody. Immunoblot analysis of ubiquitination of MDA5; Ub-K0, mutant ubiquitin with no lysine residues retained. Data are representative of three independent experiments with similar results

Next, we wondered how ARRDC4 regulated innate immune signaling pathway through MDA5. MDA5 senses viral RNA and was activated by K63 polyubiquitin chains mediating by E3 ubiquitin ligases, leading to downstream signal transduction.^11^ As ARRDC4 contains highly conserved PY motifs, which can recruit E3 ubiquitin ligases resulting in ubiquitination of target molecular,^24^ we considered whether ARRDC4 influenced K63 ubiquitination of MDA5. In response to EV71 infection, MDA5 could be K63 ubiquitinated, which was significantly impaired in ARRDC4 knockdown t-M_Ø_ (Figure 6a). In addition, we found that overexpression of ARRDC4 augmented K63 ubiquitination of MDA5 after EV71 infection, but had no effect on K0 (mutant ubiquitin with no lysine residues retained) ubiquitination of MDA5 (Figure 6b). The ARRDC4 mutant lacking ARRDC4-N (ARRDC4-ΔN), which could not interact with MDA5, was unable to enhance K63 ubiquitination of MDA5 (Figure 6c). These data suggest that ARRDC4 interacts with MDA5 and promotes K63 ubiquitination activation of MDA5 leading to innate signaling cascade during EV71 infection.

### ARRDC4 promotes MDA5 K63 ubiquitination through TRIM65

**Figure 7:**
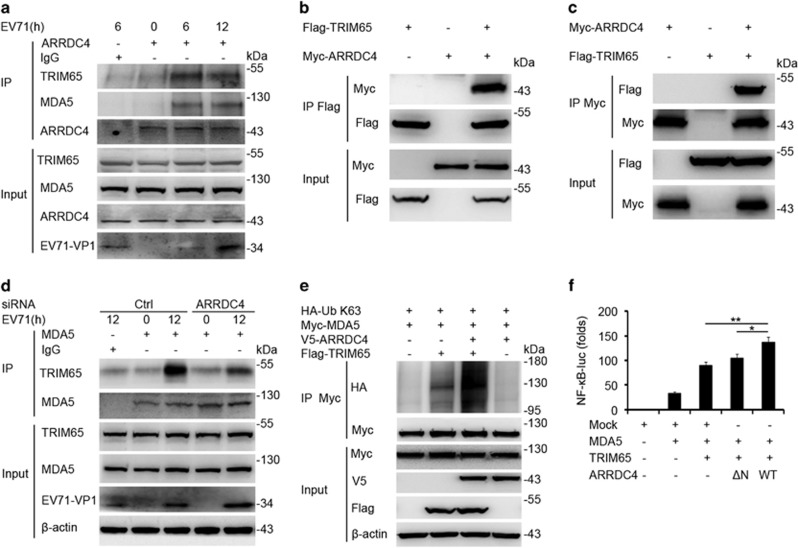
ARRDC4 promotes MDA5 K63 ubiquitination through TRIM65. (**a**) The t-M_Ø_ were infected with EV71, and IP with anti-ARRDC4 and control antibodies, followed by immunoblot analysis as indicated. (**b** and **c**) IP and immunoblot analysis of protein interaction in HEK293T cells transfected with Flag- or Myc-tagged ARRDC4 and TRIM65. (**d**) Immunoblot analysis of the association between precipitated TRIM65 and MDA5, in t-M_Ø_, which were transfected with ARRDC4 siRNA and control siRNA for 48 h and stimulated with EV71 for indicated time. (**e**) IP and immnunoblot analysis of ubiquitination of MDA5 in HEK293T cells transfected with indicated plasmids. (**f**) HEK293T cells were transfected with NF-*κ*B luciferase reporter plasmid together with the other indicated plasmids, after 48 h, the luciferase activity was determined. Mock means empty vector. Data are representative of three independent experiments with similar results (**a**-**e**), or are shown as mean±S.D. of triplicate samples, *P<0.05, **P<0.01 (**f**)

Finally, we would like to search for the E3 ubiquitin ligase, which was associated with ARRDC4 to promote K63 ubiquitination of MDA5 upon EV71 infection. It has been reported recently that TRIM65 specifically interacts with MDA5 and facilitates K63 ubiquitination of MDA5 at lysine 743, leading to oligomerization and activation of MDA5 after encephalomyocarditis virus (EMCV) infection.^28^ Thus, we further observed whether there was cooperation between TRIM65 and ARRDC4 in promoting the activation of MDA5. Through immunoprecipitation, we corroborated endogenous ARRDC4 could interact with TRIM65 and MDA5 in EV71-infected t-M_Ø_ (Figure 7a) and in HEK293T cells (data not shown). The interaction between overexpressed ARRDC4 and TRIM65 were further confirmed in HEK293T cells (Figures 7b and c). ARRDC4 silencing could reduce the interaction between TRIM65 and MDA5 in EV71-infected t-M_Ø_ (Figure 7d), suggesting that ARRDC4 may acts as the adaptor for the interaction of TRIM65 and MDA5. Furthermore, we observed that ARRDC4 only elevated low level of K63 ubiquitination of MDA5 without EV71 infection, whereas this could significantly be raised up when ARRDC4 were co-transfected with TRIM65 (Figure 7e), indicating TRIM65 was required for ARRDC4 to modulate MDA5 activation. In addition, TRIM65-catalyzed K63 ubiquitination of MDA5 was augmented by ARRDC4 (Figure 7e). ARRDC4 facilitated TRIM65- and MDA5-induced activation of NF-*κ*B luciferase reporter, however, the positive regulatory effect of ARRDC4 was disappeared without the ARRDC4-N domain (Figure 7f). These data indicate that, upon EV71 infection, ARRDC4 interacts with MDA5 and further recruits TRIM65 to mediate K63 polyubiquitin chains on MDA5, leading to downstream signaling pathways activation.

## Discussion

Upon EV71 infection, host innate immune system sets antivirus strategies to produce proinflammatory cytokines and chemokines to restrain and clear the pathogens, meanwhile, virus can also utilizes multiple factors to neutralize and escape innate immune surveillance. The underlying mechanisms of host–virus interaction during EV71 infection attract increasing concerns. We have shown here that ARRDC4 interacted with MDA5 and positively regulated the K63 polyubiquitination of MDA5 through TRIM65, leading to activation of the downstream innate immune signaling and production of cytokines, thus contributed to defense EV71 infection. Our study also confirmed the positive correlation between ARRDC4 expression level and cytokines production in clinical specimens, suggesting that ARRDC4 is increased to enhance anti-EV71 innate immunity. Some severe EV71-infected HFMD patients, who carried extremely high level of proinflammatory cytokines, were accompanied with much higher expression of ARRDC4, indicating that the aberrant increase of ARRDC4 may related to the imbalanced release of cytokines and the pathological injury during EV71 infection. We considered that ARRDC4 may serve as a potential target for moderating dysregulation of EV71-induced inflammation.

ARRDC4 is one of *α*-arrestins family members, containing arrestin-like N domain, arrestin-like C domain and highly conserved polyproline (PY) motif in the C-terminal tail. It’s known that ARRDC4 participates in glucose metabolism through inhibiting glucose uptake with its arrestin-like domains.^19, 23^ In our research, the upregulated ARRDC4 interacted with MDA5 and recruited E3 ubiquitin ligase TRIM65 for MDA5 activation, having a new function in EV71-triggered innate immune response. We identified that the arrestin-like N domain of ARRDC4 was required for the interaction with MDA5. Our data confirmed that the arrestin-like domains of arrestins family may have critical roles in mediating interaction with other molecules. The PY motif of ARRDC4 is as important as the arrestin-like domains, especially for interaction with WW domain-containing proteins, which include several ubiquitin ligases such as Nedd4.^24^ ARRDC4 binds to *β*_2_ adrenergic receptor (*β*_2_AR) and recruits Nedd4 with the PY motif to mediate ubiquitination and trafficking of *β*_2_AR.^29^ Whether ARRDC4 recruits TRIM65 through the PY motif or the other domains is still need to be uncovered. Moreover, we still wonder the mechanisms underlying ARRDC4 upregulation. It is known that the expression of ARRDC4 is upregulated by transcription factor MondoA and Mlx complex resulting in restricting glucose uptake and cell growth.^30^ We estimate that, upon EV71 infection, some transcript activators would bind to the promoter of ARRDC4 to enhance transcription of ARRDC4, and the detail mechanism will be carried out in our further research.

MDA5 has momentous roles in innate immune responses against RNA virus.^9, 10^ The K63 polyubiquitination of MDA5 is important for oligomerization and activation of MDA5,^31^ however, the regulatory mechanisms are still incomplete known. TRIM13 interacts with MDA5 and negatively regulates MDA5-mediated innate signaling upon EMCV infection,^32^ nevertheless, whether and how TRIM13 mediates ubiquitination modification of MDA5 is still elusive. RNF123 inhibits IFN-*β* production through competitively inhibiting MAVS binding to MDA5/RIG-I, however, the negative regulatory function of RNF123 is independent on its E3 ligase activity.^33^ Recently, TRIM65 was found specifically interacts with MDA5 and facilitates K63 ubiquitin chains on MDA5 at lysine 743, leading to oligomerization and activation of MDA5.^28^ Herein, we found TRIM65 was involved in EV71-induced K63 ubiquitination of MDA5, further confirmed the non-redundant roles of TRIM65 in MDA5 activation. In addition, ARRDC4 acts as the adaptor for promoting the interaction between MDA5 and TRIM65, and TRIM65 is required for ARRDC4 to modulate MDA5 activation. We provide a new regulation manner of MDA5 activation, which ARRDC4 and TRIM65 collaboratively enhance K63 ubiquitination of MDA5, further work will be put forward to explore the underlying mechanism.

During the virus life cycle, the EV71 non-structural proteins 2A and 3C have essential roles in host–virus interactions.^34^ In serum and cerebrospinal fluid of EV71-infected HFMD patients, the production of type I IFNs are impaired.^4, 5^ Accumulating evidence indicate that the 2A and 3C protein can target some crucial molecules in innate immune signaling pathways to block type I IFN production. In EV71 2A-transfected Hela cells, the 2A protein can cleave MDA5 and MAVS, leading to repression of type I IFN transcription.^35^ The activation and nuclear translocation of IRF3 were extremely inhibited by 2A and 3C proteins.^35, 36^ In the EV71-infected t-M_Ø_, we also found that the phosphorylation of IRF3 was suppressed and the production of type I IFN were undetectable. In addition, EV71 can also target type I IFN-signaling, the 2A protein induces IFNAR1 degradation,^5^ and the 3C protein cleaves IRF7^37^ and IRF9.^38^ In our study, we found that MDA5 are only partially cleaved in EV71-infected t-M_Ø_. We speculate that, once EV71 recognized by MDA5, the activation of TBK1-IRF3 signaling pathway was extremely impaired as mentioned above, however, the remained MDA5, which was not cleaved by EV71 viral proteins, could significantly activate innate signaling pathway, leading to much higher level of cytokines production. The activation of MDA5 was promoted by ARRDC4 and TRIM65, otherwise, there could be more regulation mechanisms involved in anti-EV71 innate immunity, and crosstalk between different pathways and different molecular need to be further clarified.

Collectively, we have shown that ARRDC4 interacts with MDA5 and promotes K63 polyubiquitination of MDA5 via TRIM65, consequently activates innate signaling pathway, leading to enhanced anti-EV71 immune response. Our findings suggest a new function of ARRDC4 in innate immune system, and provide new insight into the regulatory mechanism of EV71-induced inflammation, which line out a direction for intervention imbalanced inflammatory response during EV71 infection.

## Materials and Methods

### Cell culture and virus

THP-1 and HEK293T cell lines were obtained from American Type Culture Collection (Manassas, VA, USA). The base medium for THP-1 is RPMI-1640 medium (Gibco, Waltham, MA, USA) with fetal bovine serum (FBS) (Gibco, Grand Island, NY, USA) to a final concentration of 10%. The base medium for HEK293T is DMEM (Gibco, Waltham, MA, USA) with 10% FBS (Gibco, Grand Island, NY, USA). THP-1 cells were seeded into cell plates and were incubated with PMA (50nM) for 24 h, the differentiated THP-1-derived macrophages got attached to the plate bottom, then were washed with fresh media for use. The EV71/SZ50/CHN/2014 isolate (subgenotype C4a, GenBank: KT428649) used in this study was from the department of microbiology, Shenzhen Center for Disease Control and Prevention (CDC). Virus was amplified in RD cells and indentified via RT-PCR with EV71 specific primers. Viral titer was determined by TCID50 on RD cells.

### Microarray analysis

THP-1-derived macrophages were infected with EV71 for indicated time, and the total RNA was isolated using Trizol reagent (Invitrogen Corporation, Carlsbad, CA, USA), and the RNA was validated with Agilent Array platform. The microarray analysis was performed by Gminix Biotechnology Company (Shanghai, China) with Affymetrix Human Transcriptome Array 2.0 (HTA 2.0) (Affymetrix, Waltham, MA, USA). Each transcript was represented by a specific exon or splice junction probe, which can identify individual transcripts precisely.

### Study design and populations

Followed the principle of case–control study, we recruited 30 HFMD patients and 30 healthy controls in Shenzhen Children’s Hospital, Shenzhen Baoan District People’s Hospital and Shajing Institution of Disease Prevention and Healthcare from 2015 to 2016. All of the patients were diagnosed with HFMD according to the WHO Guide to Clinical Management and Public Health Response for HFMD, and were further confirmed by EV71 isolation and identification in clinical samples, such as stool, rectal and throat swabs. Patients who were treated with drugs after infection were excluded. Healthy candidates were from physical health examination children, who were matched by age, sex with the patients, and diagnosed without any infection disease and further etiological confirmed without EV71 infection. The PBMCs were isolated from the blood samples within 2 h by Ficoll paque premium from GE Healthcare Life Science (Marlborough, MA, USA) and stored in Trizol reagent at –80°C. All of the participants were informed consent and the study was approved by the Ethics Committee of the Shenzhen Center for Disease Control and Prevention.

### Reagents and antibodies

PMA were purchased from Sigma (St. Louis, MO, USA). Antibodies to ARRDC4 were from Abcam Inc. (ab209679) (Cambridge, MA, USA) or Santa Cruz Biotechnology Inc. (sc-135444) (Dallas, TX, USA). Antibodies to MDA5 (#21775-1-AP) was from Proteintech Group (Rosemont, IL, USA). Antibodies to MDA5 (D74E4, #5321), ERK (#9102), p-ERK (#9101), JNK (56G8, #9258), p-JNK (81E11, #4668), p38 (#9212), p-p38 (#9211), p65 (D14E12, #8242), p-p65 (93H1, #3033), I*κ*B*α* (L35A5, #4814), IRF3 (D83B9, #4302) and p-IRF3 (#4947) were from Cell Signaling Technology (Beverly, MA, USA). Antibody to TRIM65 (HPA021578) was from Sigma, anti-EV71-VP1 antibody (ab169442) was from Abcam Inc. The antibodies to Flag-tag (#14793), Myc-tag (#2276), HA-tag (#3724) and V5-tag (D3H8Q, #13202) were from Cell Signaling Technology. The anti-K63-ubiquitin (#05-1313) was purchased from Millipore (San Diego, CA, USA).

### Plasmids and transfection

Recombinant vectors encoding full-length and mutant human ARRDC4 (GenBank No. NM_183376.2) and IFIH1 (for MDA5) (GenBank No. NM_022168.3) were cloned into pcDNA3.1 vectors (Invitrogen Corporation) as described previously.^39^ The Flag-tagged TRIM65 constructs was provided by Dr. Rongbin Zhou (University of Science and Technology of China, Hefei, China). All constructs were confirmed by sequencing. Plasmids were transiently transfected into HEK293T cells with jetPEI reagents (Polyplus Transfection Company, Illkirch, France) following the manufacturer instructions.

### RNA interference

For transient knockdown of ARRDC4, the siRNA duplexes specific for ARRDC4 were transfected with the INTERERin according to the manufacturer protocol (Polyplus Transfection Company). The sequence of siRNA specific for human ARRDC4 was 5′-GAGAAGCUAUUCCAAUCUAUU-3′. The negative control siRNA sequence was 5′-UUCUCCGAACGUGUCACGUUU-3′. These siRNA were designed and synthesized by GenePharma Co. (Shanghai, China).

### RNA quantification

The total cellular RNA was extracted with Trizol reagent (Invitrogen Corporation), and the cDNA synthesis and Quantitative real-time PCR were performed as described previously.^39^ The primers used for ARRDC4 were 5′-GCAGGAAAGAGTCGCCCG-3′ (sense), 5′-TAAACTTGTGCGGCAATCCTG-3′ (antisense). The others specific primers for Q-PCR assay were, IL-6 forward 5′-AAATTCGGTACATCCTCGACGG-3′, IL-6 reverse 5′-GGAAGGTTCAGGTTGTTTTCTGC-3′ TNF-*α* forward 5′-ATGAGCACTGAAAGCATGATCC-3′, TNF-*α* reverse 5′-GAGGGCTGATTAGAGAGAGGTC-3′ CCL3 forward 5′-AGTTCTCTGCATCACTTGCTG-3′, CCL3 reverse 5′-CGGCTTCGCTTGGTTAGGAA-3′. *β*-Actin was used as internal quantitative control.

### Immunoprecipitation and immunoblot analysis

Total proteins of cells were prepared using cell lysis buffer and protease inhibitor mixture, then quantified the concentrations with the bicinchoninic acid assay BCA (Pierce, Rockford, IL, USA). Equal amount of proteins were used for immunoprecipitation and immunoblot analysis as described.^39^ For Flag-tag protein immunoprecipitation, anti-Flag M2 affinity gel (Sigma) was used and performed follow the technical procedure.

### Luciferase reporter assay

HEK293T cells were co-transfected with luciferase reporter plasmid, pRL-TK-Renilla luciferase plasmid and other constructs as indicated in the context. Total amounts of plasmid DNA were equalized with empty control vector. Luciferase activities were measured with the Dual Luciferase Reporter Assay System (Promega, Madison, WI, USA) according to the manufacturer’s instructions. Data are normalized for transfection efficiency by dividing firefly luciferase activity with activity of Renilla luciferase.

### Statistical analysis

The statistical significance of comparisons between two groups was determined with two-tailed Student’s *t*-test. Categorical characteristics were examined by the *χ*^2^ test. Correlation between ARRDC4 expression level and cytokines production level in clinical specimens were analyzed using Spearman correlation test. All statistical analysis performed using SPSS 17.0 (Chicago, IL, USA). *P-*values of <0.05 were considered statistically significant.
