# Supplementary material for: ARRDC4 regulates enterovirus 71-induced innate immune response by promoting K63 polyubiquitination of MDA5 through TRIM65
Source: Cell Death Dis. 2017 Jun 8;8(6):e2866–. doi: 10.1038/cddis.2017.257 (PMC5520913; doi:10.1038/cddis.2017.257)
Supplement: Supplementary Information [file cddis2017257x1.docx]

**Supplementary Information**

ARRDC4 regulates Enterovirus 71-induced innate immune response by promoting K63 polyubiquitination of MDA5 through TRIM65

Jun Meng, Zhenyu Yao, Yaqing He, Renli Zhang, Yanwei Zhang, Xiangjie Yao, Hong Yang, Long Chen, Zhen Zhang, Hailong Zhang, Xueqin Bao, Gang Hu, Tangchun Wu, Jinquan Cheng

MDA5 is most indispensable to the activation of EV71-induced innate signaling pathway, while the TLR3/7/8 may partially play some assistant roles. ARRDC4 promotes EV71-induced inflammatory cytokines production through positively regulation of MDA5-triggered innate signaling activation.


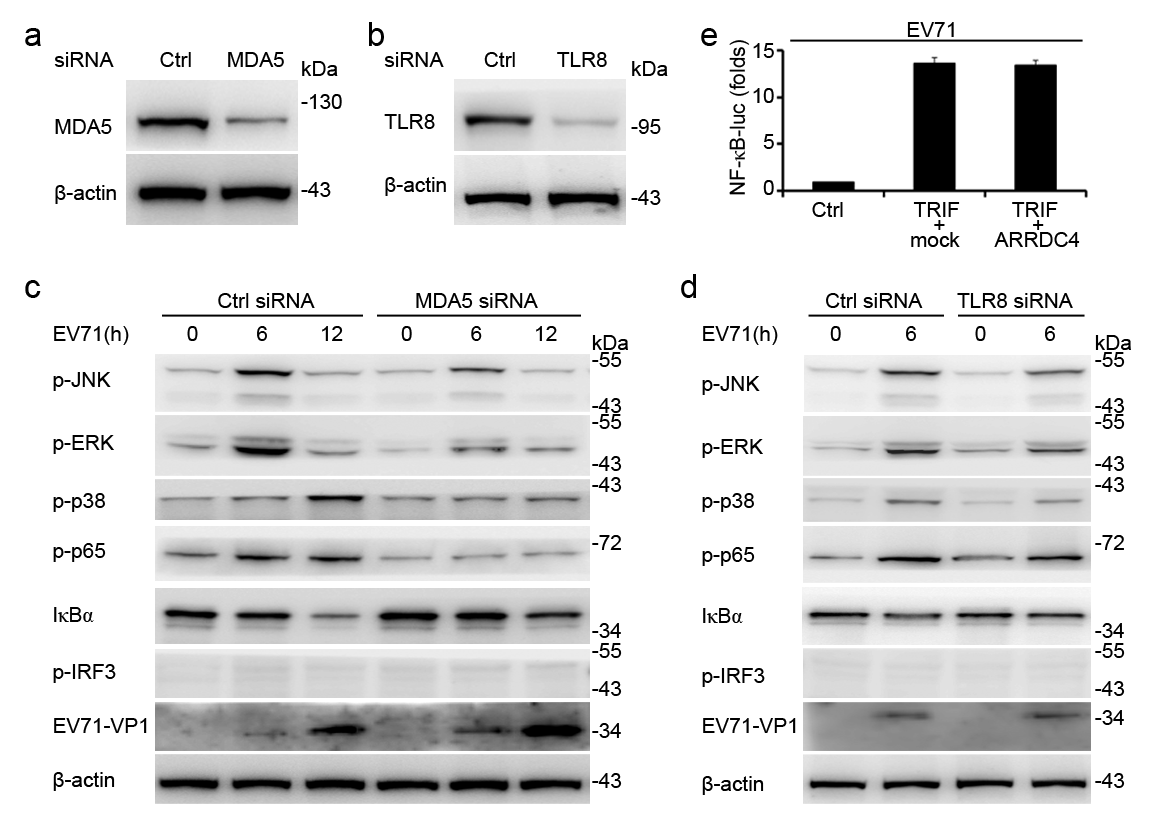


**Figure S1, MDA5 is the most indispensable PRRs for the activation of innate signaling pathway upon EV71 infection.** (**a and b**) Immunoblot analysis of MDA5 and TLR8 knockdown efficiency in protein levels in t-M_Ø_ 48h after transfected with control siRNA or MDA5siRNA (a), or TLR8 siRNA (b). (**c and d**) The t-M_Ø_ were transfected with MDA5 specific siRNA (c) or TLR8 specific siRNA (d) or the negative control siRNA. After 48h, the cells were infected with EV71 for indicated time, then phosphor-JNK, -ERK, -p38, -p65, -IRF3 and total IκBα were detected with immunoblot, the expression of EV71-VP1 and β-actin were detected as well. (**e**) HEK293T cells were transfected with NF-κB luciferase reporter plasmid together with the other indicated plasmids, after 48h, stimulated with EV71 for 12h, the luciferase activity was determined. Mock means empty vector. Data are representative of three independent experiments with similar results (a-d), or are shown as mean±SD of triplicate samples (e).
